# Supplementary material for: MG132‐induced progerin clearance is mediated by autophagy activation and splicing regulation
Source: EMBO Mol Med. 2017 Jul 3;9(9):1294–313. doi: 10.15252/emmm.201607315 (PMC5582415; doi:10.15252/emmm.201607315)
Supplement: Supplementary file 1 — Appendix [file EMMM-9-1294-s001.pdf]

MG132-induced progerin clearance is mediated by autophagy activation and splicing regulation

Karim Harhouri [1], Claire Navarro [1], Danielle Depetris [1], Marie-Geneviève Mattei [1], Xavier Nissan [2], Pierre Cau [1,3], Annachiara De Sandre-Giovannoli [1,4], Nicolas Lévy [1,4,*]

Appendix

Appendix Figure S1

Appendix Table S1

Appendix Figure S1

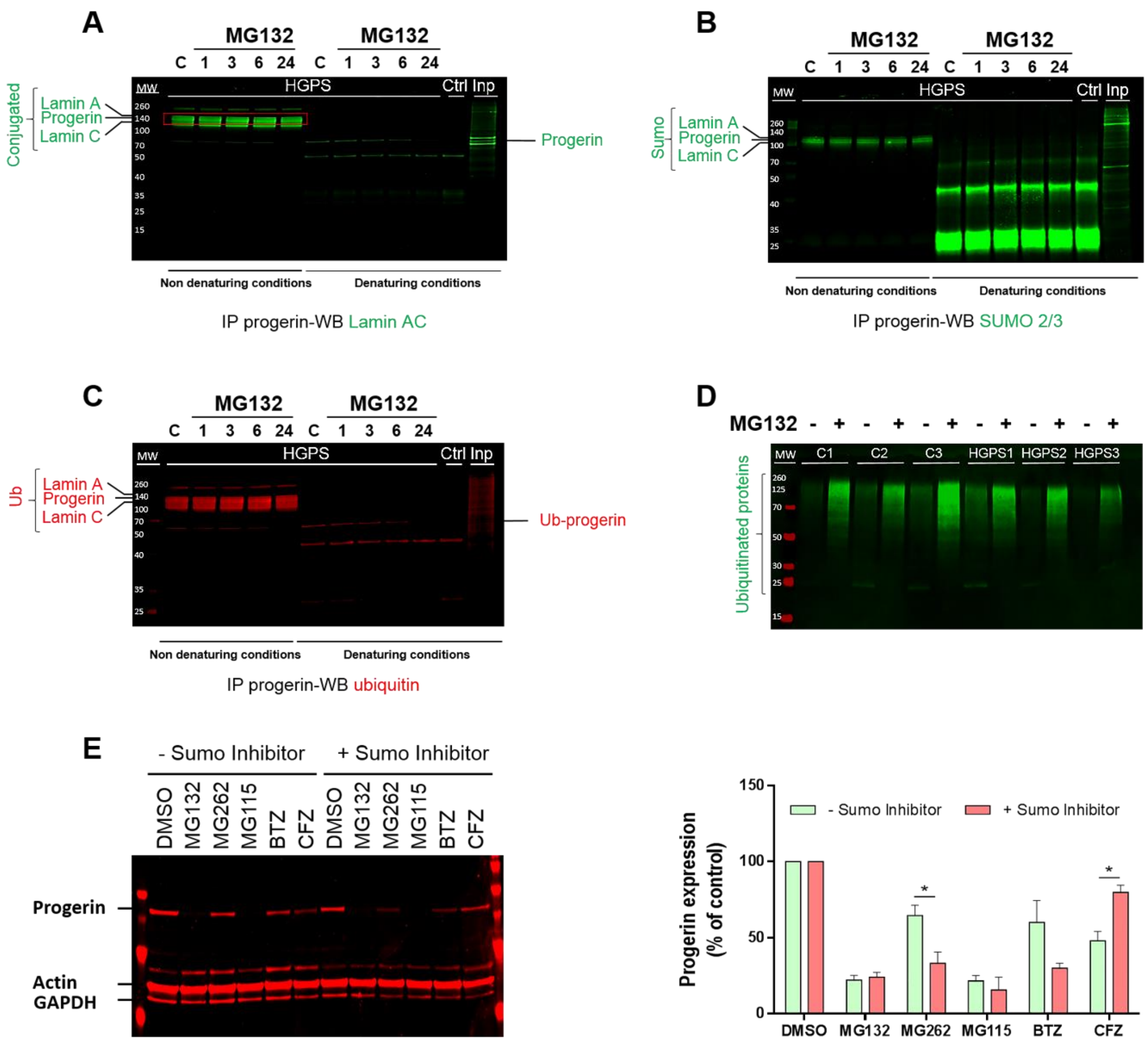

Appendix Figure S1 - Progerin sumoylation and ubiquitination.

A-C Non-denaturing and denaturing immunoprecipitation (IP) of progerin in HGPS fibroblasts treated for 1, 3, 6 and 24h with 5 µM MG132 or with vehicle control (DMSO) “C” for 24h. The same IP was performed on primary fibroblasts from healthy control individual (Ctrl). inp: input is shown in right panel.

A Treated HGPS fibroblasts were collected and then subjected to IP with anti-progerin antibody followed by western blot analysis with anti-lamin A/C antibody showing conjugated and non-conjugated lamin A/C.

B Sumoylated progerin is not susceptible to MG132 treatment. IP with anti-progerin antibody followed by western blot analysis with anti-SUMO 2/3 antibody showing sumoylated lamin A/C.

C Ubiquitinated Progerin is susceptible to MG132 treatment. IP with anti-progerin antibody followed by western blot analysis with anti-ubiquitin antibody.

D Primary dermal fibroblasts from healthy control individuals (C1, C2 and C3) and HGPS (HGPS1, HGPS2 and HGPS3) were treated with 5 µM MG132 (+) or DMSO (-) for 24 h before lysis. Ubiquitinated proteins were analyzed by immunoblotting with an anti-ubiquitin antibody.

E Left panels: Progerin, actin and GAPDH expressions in whole lysates from HGPS fibroblasts treated with or without an inhibitor of protein sumoylation (100 µM of either Ginkgolic acid or 2-D08 giving the same result). 24h later cells were treated for 48h with DMSO control or with 5 µM MG132, MG262, MG115, bortezomib (BTZ) or carfilzomib (CFZ). Right panels: Progerin expression levels relative to DMSO-treated cells were normalized to actin values using ImageJ software. (n=6).

Data information: All experiments were performed in triplicate. Results in E are expressed as mean ± SEM, Student's t-test, **p < .01); the exact P-values are indicated in Appendix Table S1.

Appendix Table S1

Figure	Samples	p-Value
**Figure 1**		
1C	MG132 3h	0,4067
	MG132 6h	0,0273
	MG132 24h	0,0034
	MG132 48h	0,0007
1D	**Progerin**	
	MG132 36h	0,0187
	MG132 48h	0,0003
	MG132+ Chloroquine 1µM	0,0008
	MG132+ Chloroquine 10µM	0,0044
	MG132+ Chloroquine 25µM	0,0011
	MG132+ Chloroquine 50µM	0,0171
1D	**LC3BII/LC3BI**	
	MG132 36h	0,0125
	MG132 48h	0,0004
	MG132+ Chloroquine 1µM	0,0001
	MG132+ Chloroquine 10µM	0,0004
	MG132+ Chloroquine 25µM	0,0007
	MG132+ Chloroquine 50µM	0,0009
	Progerin 48h MG132 vs MG132+ Chloroquine 1µM	0,8966
	Progerin 48h MG132 vs MG132+ Chloroquine 10µM	0,4709
	Progerin 48h MG132 vs MG132+ Chloroquine 25µM	0,0053
	Progerin 48h MG132 vs MG132+ Chloroquine 50µM	0,0098
**Figure 4**		
4A	Well N° 2	0,0001
	Well N°3	0,097
	Well N°4	0,0294
	Well N°5	0,0169
	Well N°6	0,0028
	Well N°7	0,4476
	Well N°8	0,005
	Well N°9	0,0233
	Well N°10	0,519
	Well N°11	0,0008

	Well N°12	0,0233
4B	MG132 6h	0,0048
	MG132 24h	0,0016
	MG132 48h	0,0014
	MG132 72h	0,0032
	MG132 96h	0,0424
4C	**Progerin**	
	MG132 6h	0,0124
	MG132 24h	0,041
	MG132 48h	0,0026
	MG132 72h	0,0557
	MG132 96h	0,0532
4C	**SRSF-1**	
	MG132 6h	0,4057
	MG132 24h	0,0025
	MG132 48h	0,0118
	MG132 72h	0,0511
	MG132 96h	0,1502
4D	**Trypsin**	
	MG132 6h	0,4228
	MG132 24h	0,0406
	MG132 48h	0,0006
	MG132 72h	0,8002
	MG132 96h	0,8443
4D	**Chymotrypsin**	
	MG132 6h	0,007
	MG132 24h	0,0016
	MG132 48h	0,0009
	MG132 72h	0,1167
	MG132 96h	0,3921
4D	**Caspase-like**	
	MG132 6h	0,023
	MG132 24h	0,0083
	MG132 48h	0,0008
	MG132 72h	0,3266
	MG132 96h	0,7869
4E	**Progerin**	
	MG132 2h	0,0545
	MG132 4h	0,0593

	MG132 6h	0,0103
	MG132 12h	0,009
	MG132 24h	0,002
	MG132 48h	0,0009
	MG132 72h	0,0007
	MG132 96h	0,0003
4E	**SRSF-5**	
	MG132 2h	0,6041
	MG132 4h	0,0003
	MG132 6h	0,0004
	MG132 12h	0,0001
	MG132 24h	0,4466
	MG132 48h	0,5127
	MG132 72h	0,855
	MG132 96h	0,317
4E	**SRSF-1**	
	MG132 2h	0,4818
	MG132 4h	0,5229
	MG132 6h	0,4188
	MG132 12h	0,6993
	MG132 24h	0,002
	MG132 48h	0,008
	MG132 72h	0,0002
	MG132 96h	0,0006
4E	**LC3BII/LC3BI**	
	MG132 2h	0,0004
	MG132 4h	0,0001
	MG132 6h	0,0002
	MG132 12h	0,0004
	MG132 24h	0,0002
	MG132 48h	0,0005
	MG132 72h	0,0004
	MG132 96h	0,0007
4F	**Progerin**	
	Control siRNA (-MG132) vs Control siRNA (+MG132)	0,0015
	Control siRNA (-MG132) vs SRSF1 siRNA (-MG132)	0,0147
	Control siRNA (+MG132) vs SRSF1 siRNA (+MG132)	0,0313
4G	**Progerin**	
	Well N°2	0,0079
	Well N°3	0,051
	Well N°4	0,3323

	Well N°5	0,8475
	Well N°6	0,0264
	Well N°7	0,0255
	Well N°8	0,0133
	Well N°9	0,7612
	Well N°2 vs Well N°6	0,0054
	Well N°2 vs Well N°7	0,0055
	Well N°6 vs Well N°9	0,0225
4G	**SRSF-1**	
	Well N°2	0,0357
	Well N°3	0,4263
	Well N°4	0,168
	Well N°5	0,0502
	Well N°6	0,0221
	Well N°7	0,0239
	Well N°8	0,5318
	Well N°9	0,8363

Figure 5

5B	MG132 vs DMSO	0,0489
5C	hgps (5y) MG132 vs DMSO	0,0154
	hgps (14y) MG132 vs DMSO	0,0337
	control (14y) MG132 vs DMSO	0,1261
	control (96y) MG132 vs DMSO	0,0216
	HGPS (14y) vs control (14y)	0,0022
	HGPS (14y) vs control (96y)	0,0029
	control (14y) vs control (96y)	0,002
5D	**Untreated vs day 0**	
	MG132 1	> 0,9999
	MG132 2	0,0066
	MG132 4	0,009
	MG132 6	0,0034
	MG132 8	0,0007
	MG132 10	0,0043
	**DMSO vs day 0**	
	MG132 1	0,2236
	MG132 2	0,0056
	MG132 4	0,004

	MG132 6	0,001
	MG132 8	0,0004
	MG132 10	0,0002
	**MG132 vs DMSO**	
	MG132 1	0,6253
	MG132 2	0,0236
	MG132 4	0,0132
	MG132 6	0,0137
	MG132 8	0,0112
	MG132 10	0,0128
5E	**MG132 vs DMSO**	
	MG132 1	0,1694
	MG132 2	0,0795
	MG132 4	0,0187
	MG132 6	0,009
	MG132 8	0,0016
	MG132 10	0,008
5F	**MG132 vs DMSO**	
	MG132 1	0,0638
	MG132 2	0,2502
	MG132 4	0,0529
	MG132 6	0,5027
	MG132 8	0,0115
	MG132 10	0,0481
5G	**MG132 vs DMSO**	
	MG132 1	0,0202
	MG132 2	0,1345
	MG132 4	0,273
	MG132 6	0,3526
	MG132 8	0,6349
	MG132 10	0,2665

Figure 7

7B	**MG132 vs DMSO**	
	Lamin A	0,435
	Progerin	0,0433
	Lamin C	0,2401
	SRSF-1	0,0153
	**MG132 vs DMSO**	

7C	Lamin A	0,2399
	Progerin	0,0039
	Lamin C	0,0112
	SRSF-1	0,0143

Figure EV1

EV1B	**Normal PML-NBs: HGPS vs control**	
	Passage 9	0,3462
	Passage 10	0,3266
	Passage 11	0,4939
	Passage 12	0,4648
	Passage 13	0,075
	Passage 14	0,0088
	Passage 15	0,0864
	Passage 16	0,0942
	Passage 17	0,0155
	Passage 18	0,0112
	Passage 19	0,0112
	Passage 20	0,0047
	Passage 21	0,0119
	Passage 22	0,0289
	Passage 24	0,0043
	Passage 25	0,0013
	Passage 26	0,0009
	**Abnormal PML-NBs: HGPS vs control**	
	Passage 9	0,3462
	Passage 10	0,3266
	Passage 11	0,4939
	Passage 12	0,4648
	Passage 13	0,075
	Passage 14	0,042
	Passage 15	0,1124
	Passage 16	0,0942
	Passage 17	0,0155
	Passage 18	0,0143
	Passage 19	0,0255
	Passage 20	0,0047
	Passage 21	0,0119
	Passage 22	0,0211
	Passage 24	0,002
	Passage 25	0,004
	Passage 26	0,0008

Figure EV3

EV3C	**MG132 vs DMSO**	
	MG132	0,0008
	MG115	0,0011
	MG262	0,0008
	BTZ	0,0035
	CFZ	0,002
EV3D	**MG132 vs DMSO**	
	Chymotrypsin	0,0001
	Trypsin	0,0002
	Caspase-like	0,0001
	**Bortezomib vs DMSO**	
	Chymotrypsin	0,0001
	Trypsin	0,0001
	Caspase-like	0,0002
	**Carfilzomib vs DMSO**	
	Chymotrypsin	0,0001
	Trypsin	0,004
	Caspase-like	0,0001

Figure EV4

EV4A	**Lamin A: MG132 vs DMSO**	
	MG132 1h	0,0364
	MG132 3h	0,0804
	MG132 6h	0,0194
	MG132 24h	0,0046
	MG132 48h	0,0014
	**Lamin C: MG132 vs DMSO**	
	MG132 1h	0,4152
	MG132 3h	0,458
	MG132 6h	0,007
	MG132 24h	0,0009
	MG132 48h	0,0023
EV4B	**Lamin A: MG132 vs DMSO**	
	MG132 6h	0,016
	MG132 24h	0,0241
	MG132 36h	0,0026

	MG132 48h	0,0438
	**Lamin C: MG132 vs DMSO**	
	MG132 6h	0,0392
	MG132 24h	0,001
	MG132 36h	0,0218
	MG132 48h	0,0439
**Appendix Figure S1E**	**+Sumo Inhibitor vs -Sumo Inhibitor**	
	MG132	0,6888
	MG262	0,0351
	MG115	0,5407
	BTZ	0,1099
	CFZ	0,0145
