# Supplementary material for: MG132‐induced progerin clearance is mediated by autophagy activation and splicing regulation
Source: EMBO Mol Med. 2017 Jul 3;9(9):1294–313. doi: 10.15252/emmm.201607315 (PMC5582415; doi:10.15252/emmm.201607315)
Supplement: Supplementary file 2 — Expanded View Figures PDF [file EMMM-9-1294-s002.pdf]

Expanded View Figures

Figure EV1. Identification of thread-like PML-NBs, progerin-accumulating compartments, as novel biomarkers in late passage HGPS cell lines.

A Progerin accumulation in the nuclei of HGPS fibroblasts at successive passages. Immunofluorescence microscopy on primary dermal fibroblasts from an individual with HGPS and a healthy individual (control) stained with DAPI (blue) and progerin (red).

B Left panel, labeling of PML in the nucleus of HGPS and control (healthy individual) fibroblasts at successive passages. Right panel, abnormal PML-NBs (ring-like and thread-like: arrows) structures increase in HGPS fibroblasts nuclei vs. control fibroblasts. The experiments were performed on fibroblasts of HGPS patients and healthy subjects matched for age and passage.

C Upper panel: Progerin accumulates and localizes in PML-NBs. Immunofluorescence microscopy on primary dermal fibroblasts from an individual with HGPS and a healthy individual (control) stained with DAPI (blue), progerin (red), and PML (green). Lower panel: confocal images of lateral (xy) and axial (xz) sections of an HGPS fibroblast nucleus. Cells were stained with DAPI (blue), progerin antibody (red), and PML antibody (green).

D Ring-like and thread-like PML-NBs in HGPS fibroblast nuclei contain the main components of conventional PML-NBs including PML, SP100, HP-1, ATRX, DAXX, and CBP.

E Left panel: confocal cross section in XY and XZ of HGPS fibroblast nucleus stained with TO-PRO3 (blue) and PML (green); curves are the intensities plots along the red lines. Right panel: visualization of rotated planes of a 3D-volume view of PML (green) within the nucleus. The blue nuclear counterstain TOPRO-3 is rendered more or less transparent. Confocal stacks were recreated in three dimensions with Imaris software. Lower panel: immunofluorescence microscopy on primary dermal fibroblasts from an individual with HGPS and a healthy individual (control) stained with DAPI (blue), PML (green), and endoplasmic reticulum marker calreticulin (red).

Data information: Experiment results in (B) are expressed as mean ± SEM, $n = 4$, Student's t-test, $^{*}P < 0.05$, $^{**}P < 0.01$; the exact P-values are indicated in Appendix Table S1. Experiments in (A–E) were performed on four different cell lines showing the same results. Scale bars, 5 μm.

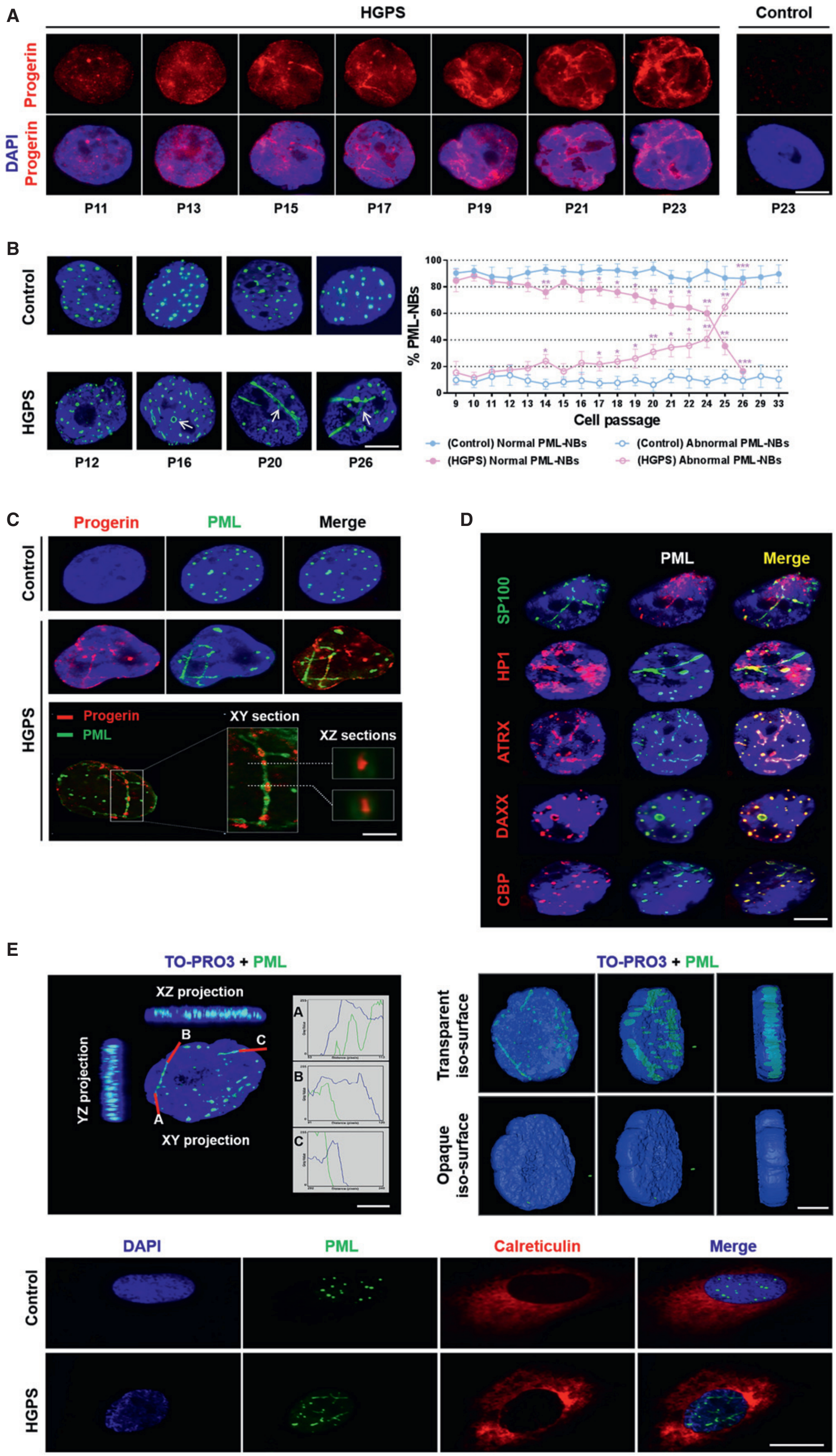

Figure EV1.

 © 2017 The Authors

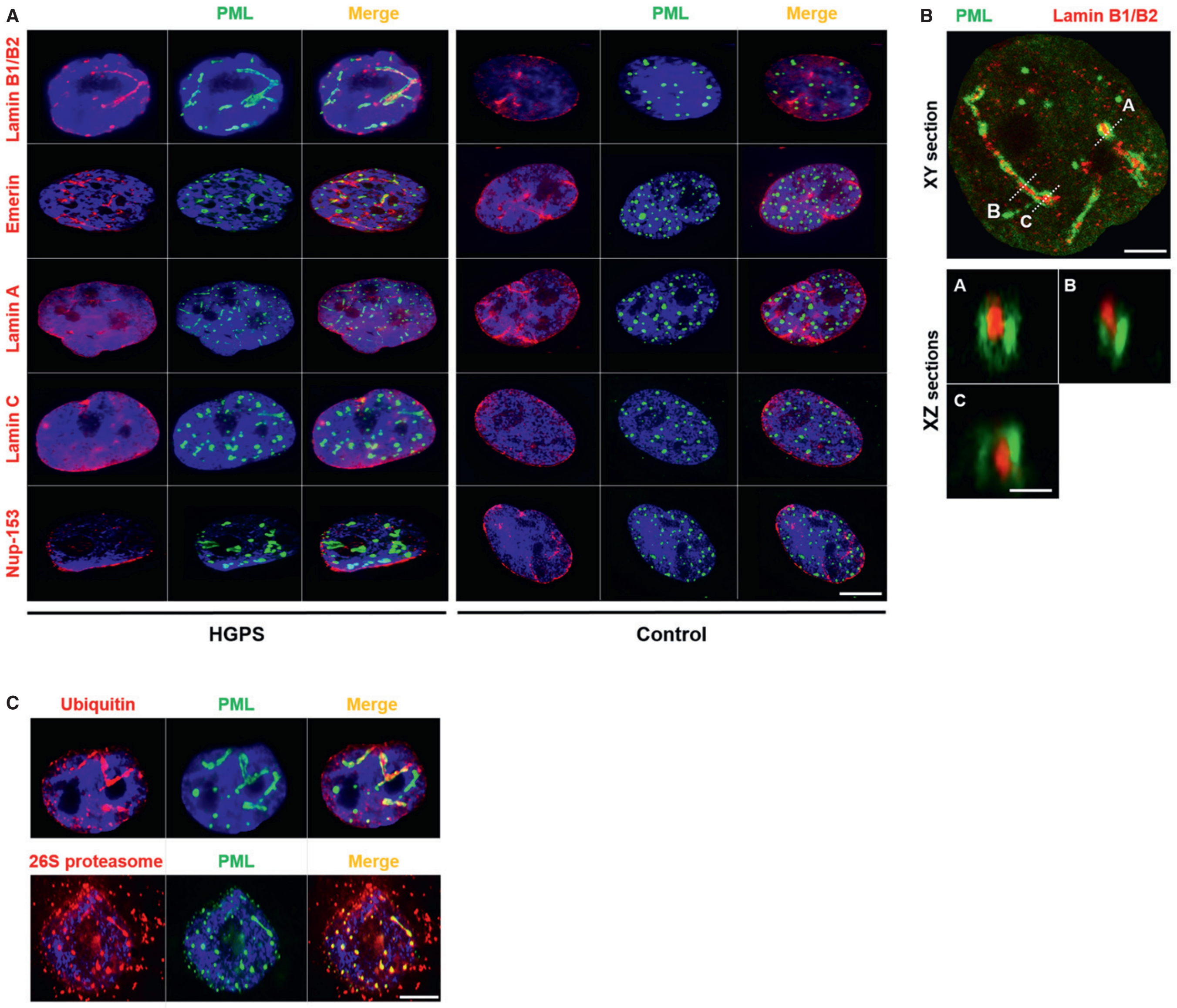

Figure EV2. Lamin B1/B2, emerin, ubiquitin, and 26S proteasome but neither lamin A/C nor Nup153 is included within PML-NBs in HGPS fibroblasts.

A Colocalization of lamin B1/B2 and emerin with PML in HGPS fibroblast nuclei. Immunofluorescence images of primary dermal fibroblasts from an individual with HGPS (left panels) and a healthy individual (control) (right panels) stained with DAPI (blue), PML (green), and antibodies to lamin B1/B2, emerin, lamin A, lamin C, and Nup-153 (red). The merged images are shown. There are no PML-NBs labeling with lamin A (red), lamin C (red), or Nup-153 (red) antibodies. Scale bar, 5 μm.

B Confocal images of lateral (XY) sections (upper panel; scale bar, 2 μm) and axial (XZ) sections along the A, B, and C axis (lower panels; scale bar, 400 nm) of an HGPS fibroblast nucleus stained with lamin B1/B2 (red) and PML (green) antibodies.

C Immunofluorescence images showing colocalization of ubiquitin (red) and 26S proteasome (red) with PML (green) in the nucleus of HGPS fibroblasts. Nucleus was labeled with DAPI (blue). The merged images are shown. Scale bar, 5 μm.

Data information: Experiments in (A–C) were performed on four different cell lines showing the same results.

© 2017 The Authors

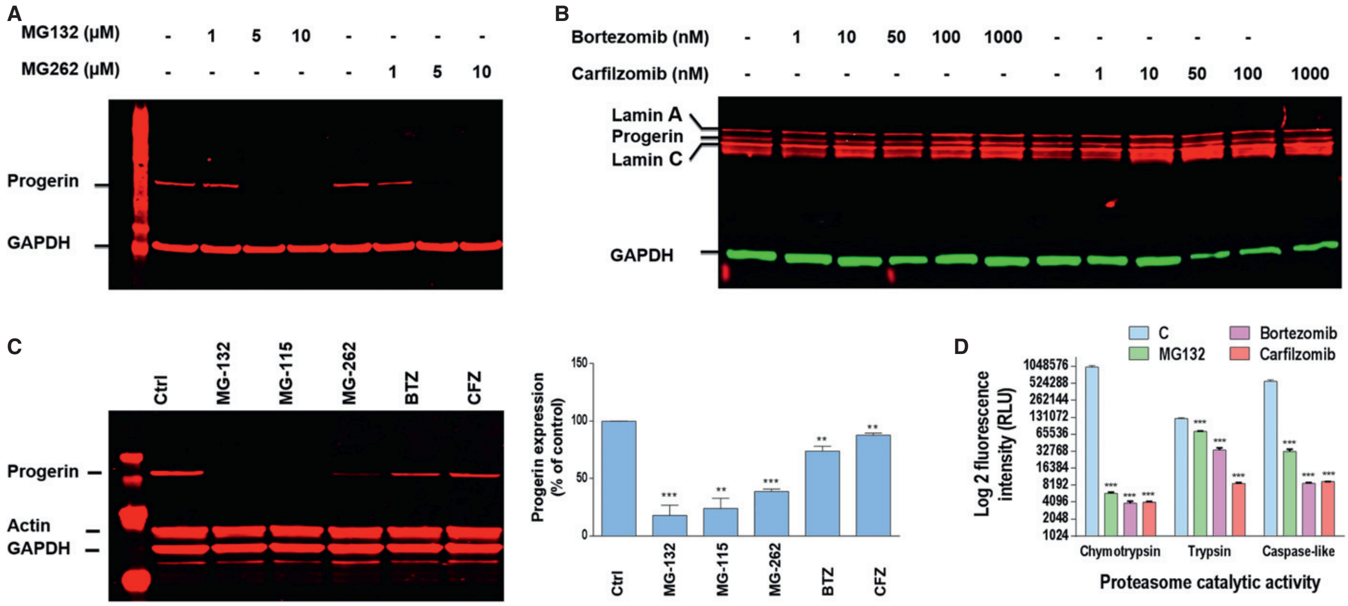

Figure EV3. Peptide aldehyde proteasome inhibitor (MG132, MG115), and peptide boronate (MG262) but not other analogs (bortezomib and carfilzomib) elicited efficient progerin clearance.

- A MG132 and MG262 induced clearance of progerin. A representative Western blotting experiment in whole lysates of HGPS fibroblasts showing progerin and GAPDH expression in MG132- and MG262-treated HGPS cells at the indicated concentrations relative to DMSO-treated (—) cells for 24 h (Control). (n = 5).
- B Western blotting evaluation of lamin A/C, progerin and GAPDH in whole lysates from HGPS fibroblasts untreated (—) or treated with bortezomib or carfilzomib for 24 h at indicated concentrations. (n = 4).
- C Left panels: progerin, actin, and GAPDH expressions in whole lysates from HGPS fibroblasts treated for 48 h with DMSO (Ctrl) or 5 μM MG132, MG115, MG262, bortezomib (BTZ), or carfilzomib (CFZ). Right panels: Progerin expression levels relative to DMSO-treated cells were normalized to actin values using ImageJ software. (n = 6).
- D Proteasome activities (trypsin: Z-LRR aminoluciferin, chymotrypsin: Suc-LLVY aminoluciferin and caspase-like: Z-nLPnLD-aminoluciferin) in HGPS fibroblasts upon 24 h MG132, bortezomib, or carfilzomib treatment, used at 5 μM. (n = 4).

Data information: Results in (C, D) are expressed as mean ± SEM, Student's t-test, $^{**}P < 0.01$, $^{***}P < 0.001$); the exact P-values are indicated in Appendix Table S1. Luminescence is determined as relative light units (RLU).

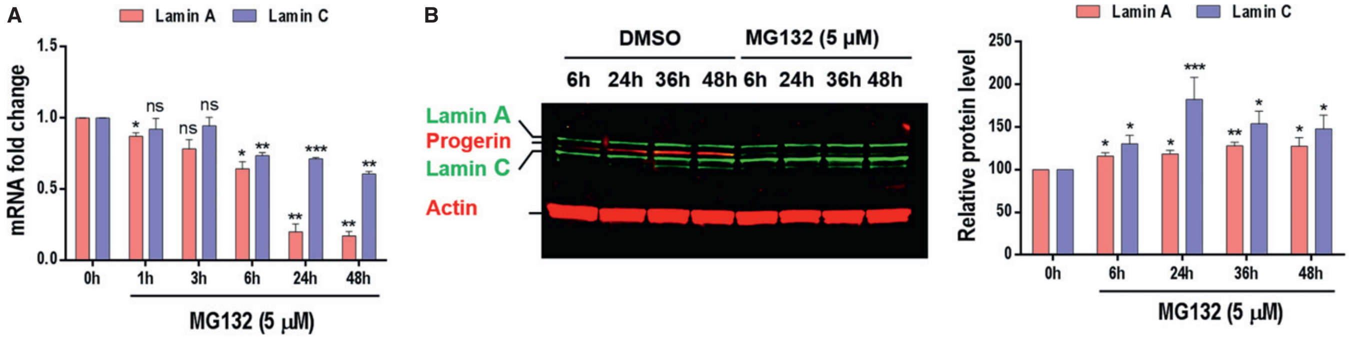

Figure EV4. MG132 treatment resulted in a decrease in the mRNA levels but not the protein levels of lamin A and C.

- A MG132 reduces lamin A and lamin C transcript levels. Quantitative real-time PCR analyses of lamin A and lamin C mRNA levels in HGPS fibroblasts treated with 5 μM MG132.
- B Both lamin A and lamin C protein levels are increased upon HGPS fibroblasts treatment with 5 μM MG132.

Data information: All experiments were performed in triplicate. Results are expressed as mean ± SEM. $^{*}P < 0.05$, Student's t-test, $^{**}P < 0.01$, $^{***}P < 0.001$, experimental vs. control (DMSO-treated cells); the exact P-values are indicated in Appendix Table S1.

 © 2017 The Authors

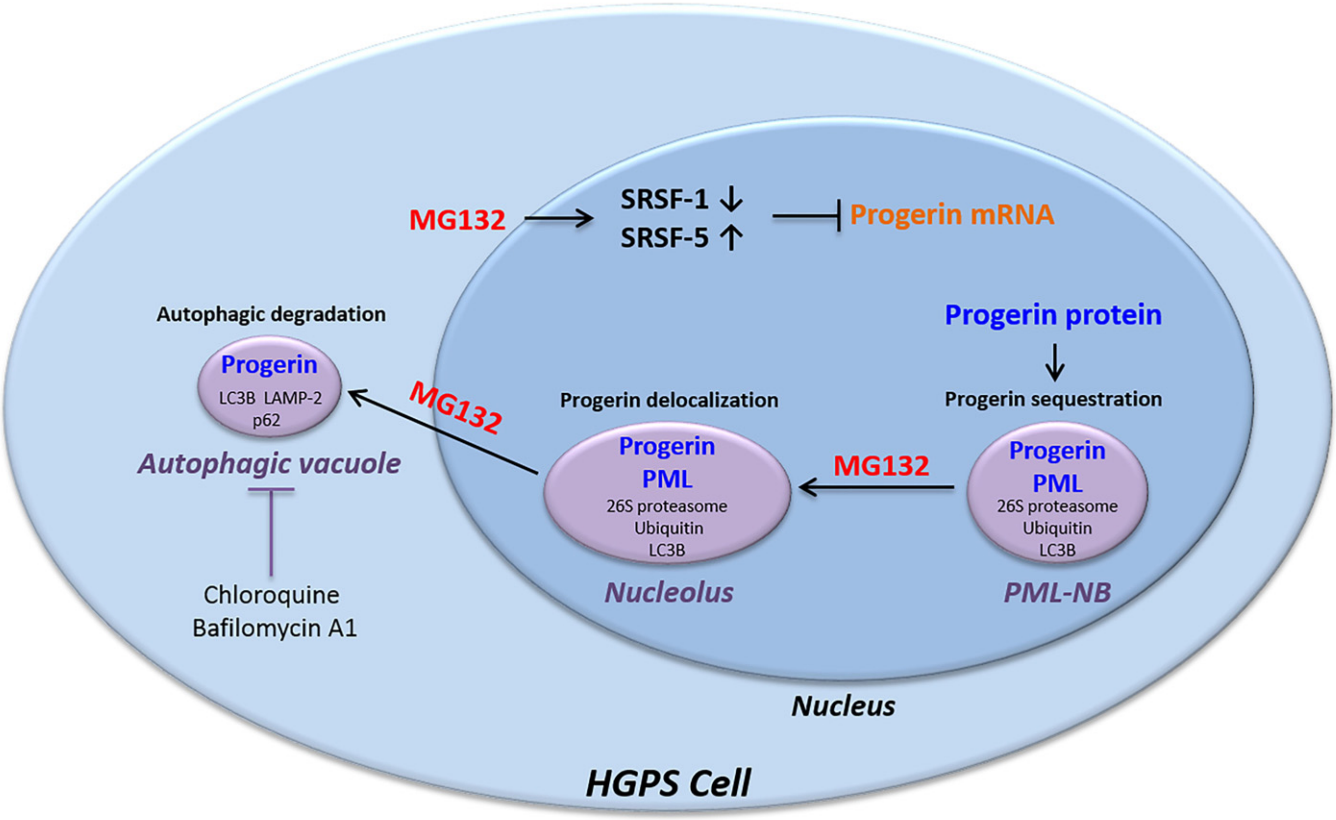

Figure EV5. Summary diagram showing MG132 effects on progerin localizations and putative involvement of caspases, splicing, and autophagy systems in progerin clearance.

In fibroblasts from HGPS patients, progerin accumulates in thread-like PML-NBs. MG132 treatment resulted in a caspase-mediated reduction of SRSF-1 levels as well as SRSF-5 accumulation, leading to a decrease in progerin transcript levels. In parallel, MG132 at first induces nucleolar translocation of progerin and then its accumulation and degradation in autophagic vacuoles.

© 2017 The Authors
