# Supplementary figures and images for: MG132‐induced progerin clearance is mediated by autophagy activation and splicing regulation
Source: EMBO Mol Med. 2017 Jul 3;9(9):1294–313. doi: 10.15252/emmm.201607315 (PMC5582415; doi:10.15252/emmm.201607315)

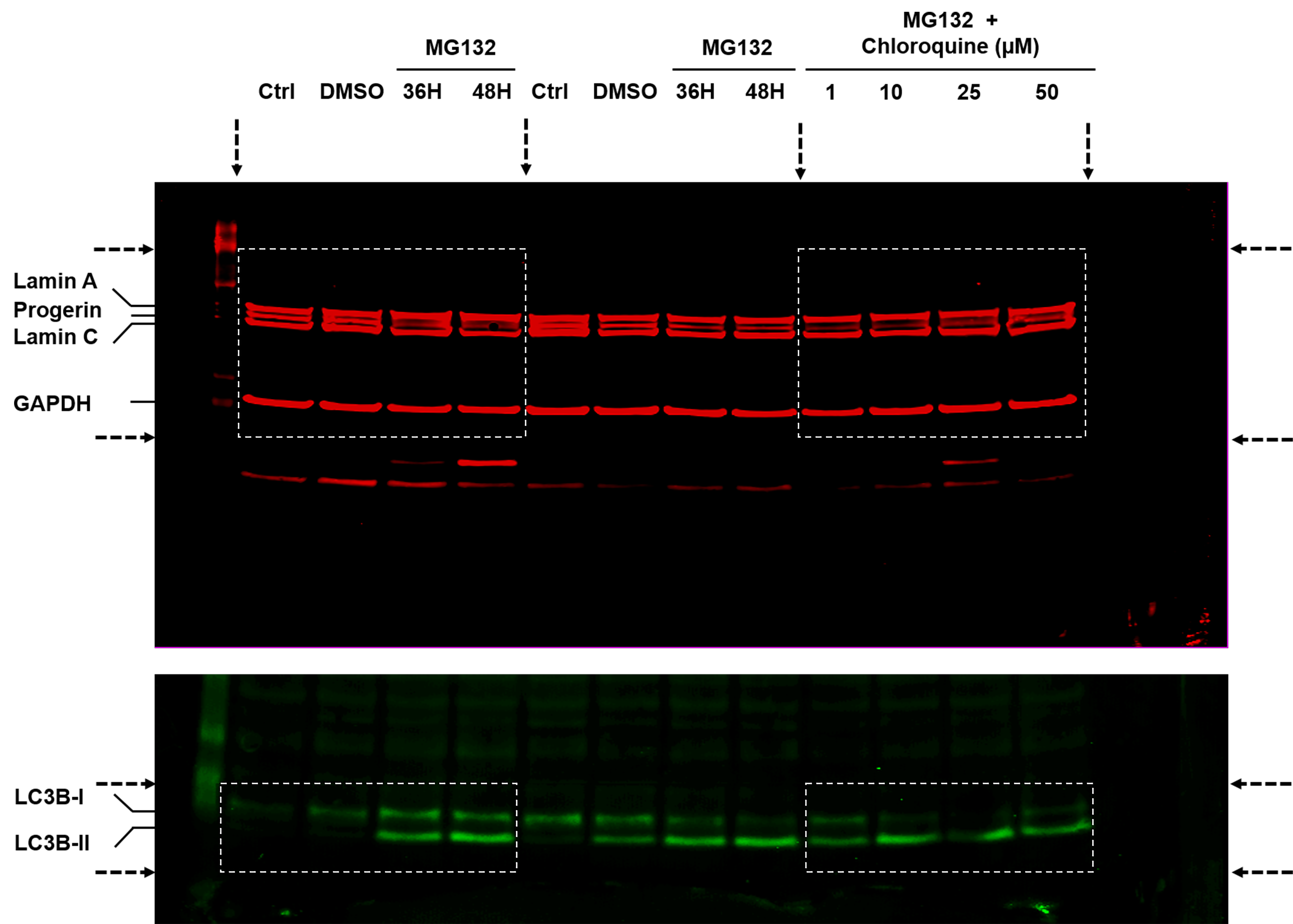

Supplement: Supplementary file 4 — Source Data for Figure 1D [file EMMM-9-1294-s003.tif]

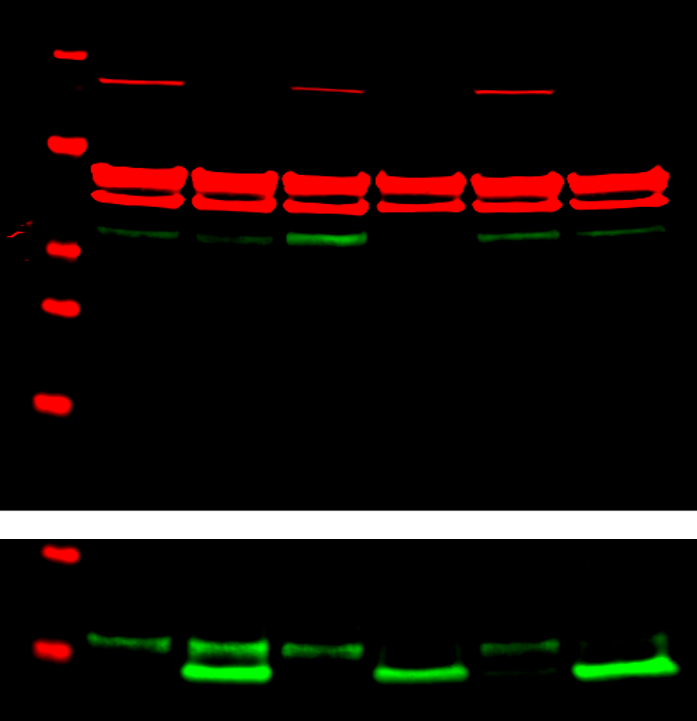

Supplement: Supplementary file 6 — Source Data for Figure 6B [file EMMM-9-1294-s005.tif]
